# Preparation of Solution Blown Polyamic Acid Nanofibers and Their Imidization into Polyimide Nanofiber Mats

**DOI:** 10.3390/nano7110395

**Published:** 2017-11-17

**Authors:** Jing Li, Guocheng Song, Junrong Yu, Yan Wang, Jing Zhu, Zuming Hu

**Affiliations:** State Key Laboratory for Modification of Chemical Fibers and Polymer Materials, College of Materials, Science and Engineering, Donghua University, Shanghai 201620, China; leeo1002@outlook.com (J.L.); sgc_34@163.com (G.S.); wy@dhu.edu.cn (Y.W.); zhj@dhu.edu.cn (J.Z.); hzm@dhu.edu.cn (Z.H.)

**Keywords:** solution blow spinning (SBS), polyamic acid, process parameters, polyimide, thermal imidization, nanofibers

## Abstract

Solution blow spinning (SBS) is an innovative process for spinning micro/nanofibers. In this paper, polyamic acid (PAA) nanofibers were fabricated via a SBS apparatus and then imidized into polyimide (PI) nanofibers via thermal process. The morphology and diameter distributions of PAA nanofibers were determined by scanning electron microscope (SEM) and Image Tool software, the processing parameters, including PAA concentration, solution feeding rate, gas pressure, nozzle size, and receiving distance were investigated in details. The fourier transform infrared spectroscopy (FTIR) was used to characterize the chemical changes in the nanofibers after thermal imidization. The results showed that the solution concentration exhibited a notable correlation with spinnability, and the formation of bead defects in PAA nanofibers. Solution feeding rate, gas pressure, nozzle size, and receiving distance affected nanofiber production efficiency and diameter distribution. The average diameters of fibers produced ranged from 129.6 to 197.7 nm by varying SBS parameters. Precisely, PAA nanofibers with good morphology were obtained and the average diameter of nanofibers was 178.2 nm with optimum process parameter. After thermal imidization, the PI nanofibers exhibited obvious adhesion morphology among interconnected fibers, with an increased average diameter of 209.1 nm. The tensile strength of resultant PI nanofiber mat was 12.95 MPa.

## 1. Introduction

Novel nanofiber techniques have gained wide interest, since nanofibers possess extraordinary structure features, novel performance, and exhibit tremendously potential application [1]. Electrospinning (ES) is the most common process used to produce fibers with micron- to nanometer-sized diameters [2]. In the process, the ultrafine fibers are generated as the suitable viscous polymeric solution is continuously stretched due to electrostatic repulsions between the surface charges and the evaporation of solvent [3,4]. Although ES has been densely adopted for continuously making polymeric nanofibers [5], some drawbacks still remain, for instance, the requirement of collector’s conductivity and the safety issue caused by high voltage, it also suffers from a relatively low fiber production efficiency, these restrictions prohibit it from being applied in industrialization [6].

The next-generation nano-manufacturing techniques will be toward high yields and processing security, as the nanofibers market is growing. For this purpose, emerging technologies have been employed, for instance, forcespinning (FS), which uses centrifugal force to process a polymer solution or polymer melt into nanofibers with the capability for mass production [7,8,9]. FS features its almost 100% yield and solvent-free process (i.e., melt forcespinning), thereby making it a more cost effective method and better safety operation when compared to other nanofiber making techniques. As a promising alternative method, solution blow spinning (SBS) is newly developed to fabricate polymeric micro/nanofibers [10,11,12]. It could produce nanofibers that are analogous to those electrospun from polymers dissolved in suitable solvents [13]. SBS process is similar to that used in conventional melt blow spinning, it only requires a simple apparatus, a homogeneous polymer solution in a volatile solvent, and a high-velocity gas source [14]. For spinning apparatus, a specialized nozzle that consists of an inner nozzle, through which a polymer solution is pumped and a concentric outer nozzle that supplies pressurized gas is of great necessity [15]. During the SBS process, the solution streams are stretched to ultrathin jets by high-velocity gas flow from outer nozzle and is solidified into nanofibers after solvent evaporation [16]. It is worth noticing that SBS is superior to ES in terms of production efficiency due to its high solution feeding rate per orifice (2.0–10.0 g/h) [17]. It is reported that SBS can produce tens of times more nanofibers than ES with an improved cost/benefit ratio [18]. Additionally, apart from restriction on the electrical polymer solution and high-voltage equipment are not required, it is easy to use as a die assembly with many nozzles without considering of electric field interference [19]. Furthermore, one can use the multi-orifice die assembly of conventional melt blow spinning to fabricate nanofibers in industrialized level via SBS.

Over the last few years, SBS has been successfully employed to produce micro/nanofibers of many polymers, including cellulose [20], polyacrylonitrile (PAN) [21], nylon-6 [22], polyvinylidene fluoride (PVDF) [3], sulfonated polyether ether ketone (SPEEK) [23], polyvinyl chloride (PVC) [24], polyvinyl alcohol (PVA) [25], poly(lactic acid) (PLA) [26], PLA/hydroxypropyl methylcellulose [27], polymethyl methacrylate (PMMA) [28], soy protein [29], and even inorganic ZnO nanofibers [16], as well as core-shell fibers with cellulose core and polyethylene oxide (PEO) shell [20]. Polyimide (PI) is a polymer of imide monomers, PI fibers are usually retrieved from their precursor polyamic acid (PAA) fibers because it is hard to melt PI or dissolve it in the solvent. PI fibers own characteristics of excellent thermal stability and flame resistance, good chemical resistance, and electrical insulation property, as well as outstanding mechanical properties [30]. Given this, PI-based nanofiber mat has been considered as a prospective material for high-temperature high-efficiency filtration and lithium battery separator, the exploration methods for rapid manufacturing PI nanofibers are of vital practical significance.

In the present work, we adopt the facile SBS method to fabricate PAA nanofibers. The processing parameters are optimized, the effects of polymer concentration, solution feeding rate, gas pressure, nozzle inner diameter, and receiving distance on the nanofibers morphology and diameter distribution of resultant PAA nanofibers are systematically evaluated by means of SEM and image tool analysis software. Afterwards, PI nanofibers are produced via thermal imidization process from as-spun PAA nanofibers. The chemical and morphological changes in the nanofibers before and after imidization are analyzed. To the best of our knowledge, this is the first comprehensive study of fabricating PAA nanofibers via the SBS process, and this work will help to show the future applicability in large-scale production of PI nanofibers.

## 2. Results and Discussion

### 2.1. SBS Parameters for PAA Solution

The polymer concentration, solution feeding rate, gas pressure, nozzle size, and receiving distance during SBS process are important factors affecting the morphology and diameter distributions of solution blown PAA nanofibers.

#### 2.1.1. The Effect of PAA Concentration

**Figure 1:**
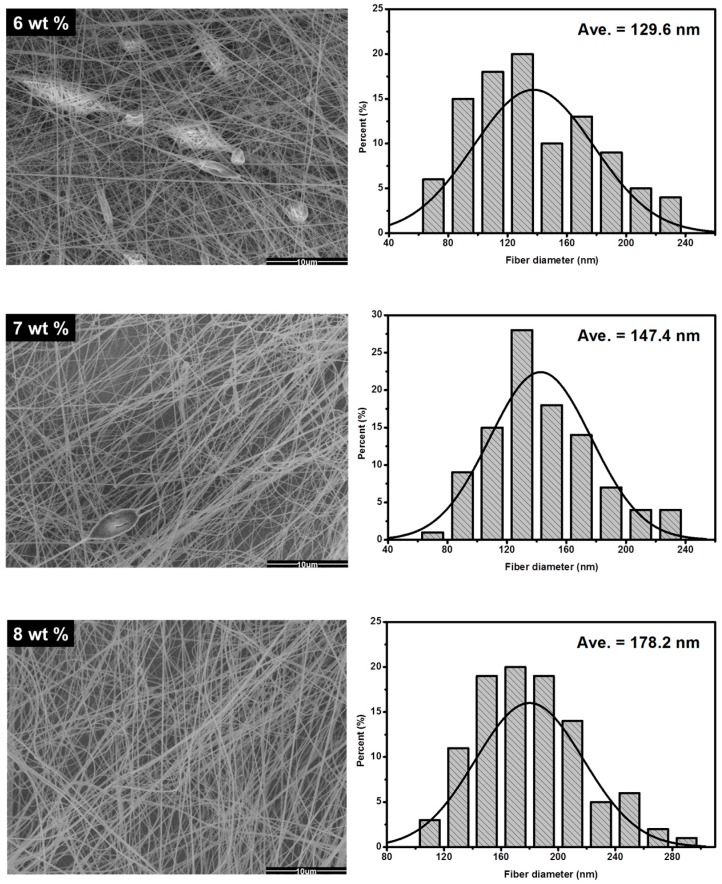
SEM (scanning electron microscope) micrographs and diameter distributions of solution blown parallel coaxial nozzles (PAA) nanofibers at different polymer concentration. The solution feeding rate, gas pressure, inner nozzle diameter and receiving distance were fixed as 2.4 mL/h, 0.08 MPa, 0.4 mm and 20 cm, respectively. (Ave. indicated the average diameter of fibers).

Concentration of polymer solution is the primary key factor affecting the spinnability in SBS process that it has to be properly controlled. Changing the concentration could alter the solution viscosity [31]. It is noteworthy that trial and error indicates that PAA nanofibers formation was relatively feasibility at a reasonable concentration range of 6–8 wt %. The excessive concentration of solutions (>8 wt %) presented difficulties in the spinning process because the nozzle was blocked frequently due to increased viscosity while concentrations below 6 wt % could result in a large number of droplets in nanofibers due to relatively low viscosity. As presented in Figure 1, the as-spun nanofibers were collected with random orientation to form a nanofiber mat. Intriguingly, three-dimensional crimping and twisted loop structure was observed due to the effect of the turbulent gas flow. Much spindle-like beads occurred at 6 wt %, and gradually decreased with an increasing PAA concentration, nanofibers with concentration of 8 wt % exhibited the optimum morphology of relatively smooth and defect-free in nanofiber mat. Trying out heating, the entire polymer solution is helpful for spinnability to reduce the solution apparent viscosity; unfortunately, it may cause polymer degradation and resultant poor fiber properties. In addition, the average diameters of PAA nanofibers were 129.6, 147.4, and 178.2 nm corresponding to PAA concentration at 6%, 7%, and 8 wt %, respectively. Obviously, the fiber diameter was positively associated with viscosity of polymer solution, increased polymer concentration produced nanofibers with larger average diameter. The surface tension effects could be dominant with increased polymer concentration/solution viscosity [32], which resulted in higher resistance to drawing forces of high-velocity gas flow [33]. Therefore, the diameter of PAA nanofibers dramatically increased with an increasing polymer concentration. When considering the solution blow spinnability and resultant nanofibers morphology, the optimal PAA concentration for SBS in this study was determined to be 8 wt %.

#### 2.1.2. The Effect of Solution Feeding Rate

**Figure 2:**
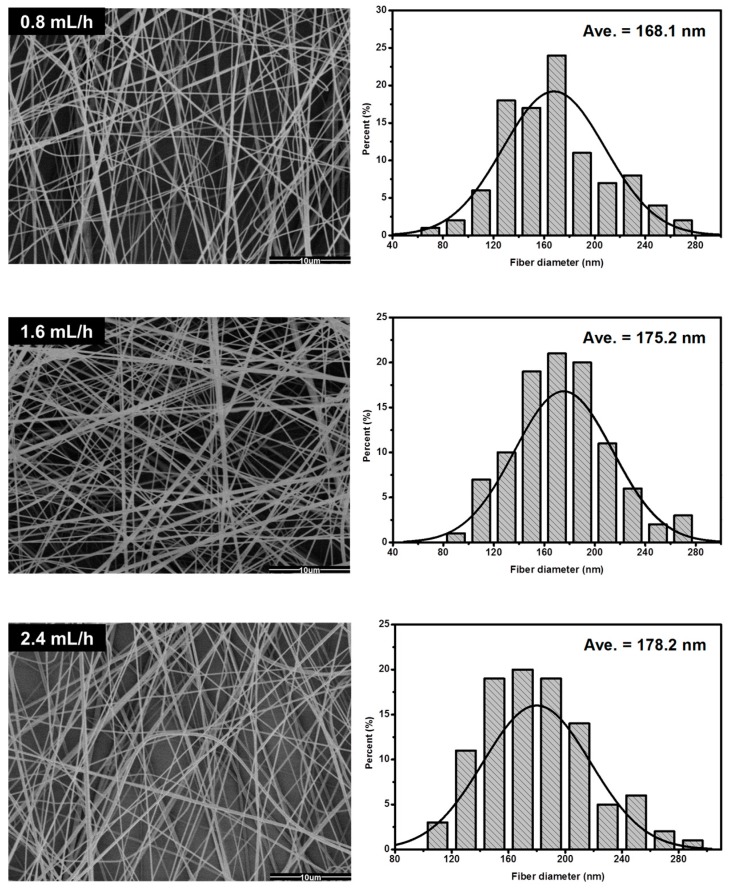

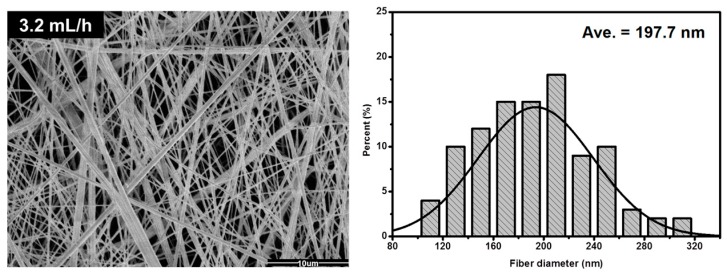
SEM micrographs and diameter distributions of solution blown PAA nanofibers at different feeding rate. PAA concentration, gas pressure, inner nozzle diameter, and receiving distance were fixed as 8 wt %, 0.08 MPa, 0.4 mm and 20 cm, respectively.

The effect of solution feeding rate (varied from 0.8 to 3.2 mL/h) on the morphology and diameter distributions of PAA nanofibers was evaluated at a fixed PAA concentration, gas pressure, inner nozzle diameter, and receiving distance of 8 wt %, 0.08 MPa, 0.4 mm and 20 cm, respectively. The results shown in Figure 2 indicated that the average fiber diameters ranged from 168.1 to 197.7 nm when the solution feeding rate increased from 0.8 to 3.2 mL/h. The overall fiber diameters increased with the increase of solution feeding rate. Moreover, further comparison indicated that the fiber diameter increased gradually when the feeding rate increased at the range of 0.8–2.4 mL/h, and the nanofibers exhibited smooth surface and with no obvious beads. When the feeding rate reached 3.2 mL/h, the fiber diameter increased significantly, accompanied by a great many doublings in the nanofibers. A possible reason for this behavior was that as the feeding rate increased, the shearing force of flowing gas applied on the surface per unit volume solution was correspondingly decreased, and the drawing velocity gradient on the extruded solution also became weaker, thus the high velocity air flow released from the outer nozzle could not sufficiently stretch the solution stream, resulting in a larger fiber diameter and adhesive interconnection between nanofibers. A high solution feeding rate produced higher fiber productivity, thus the optimal PAA solution feeding rate was selected as 2.4 mL/h in our study.

#### 2.1.3. The Effect of Gas Pressure

**Figure 3:**
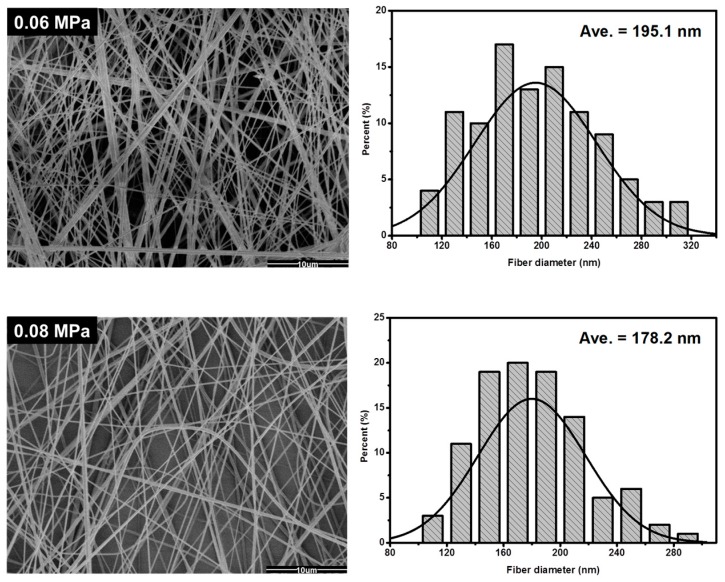

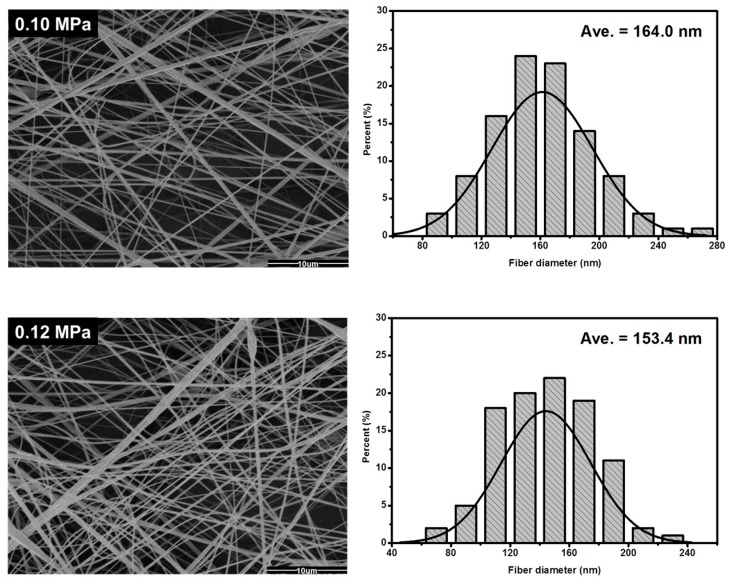
SEM micrographs and diameter distributions of solution blown PAA nanofibers at different gas pressure. PAA concentration, solution feeding rate, inner nozzle diameter and receiving distance were fixed as 8 wt %, 2.4 mL/h, 0.4 mm and 20 cm, respectively.

In the case of SBS, high velocity gas flow has the function of evaporating the solvent, deforming the solution streams, and solidifying them into the fibers. Figure 3 showed SEM morphology and fiber diameter distributions of PAA nanofibers when the gas pressure altered and other SBS parameters were fixed. As can be seen in Figure 3, the gas pressure had a linear influence on the fiber diameters. When the gas pressure increased from 0.06 to 0.12 MPa, the average diameters showed a significant decrease from 195.1 nm to about 153.4 nm, correspondingly. In addition, beads or droplets were almost absent in throughout nanofiber mat, with the optimal PAA concentration of 8 wt % and feeding rate of 2.4 mL/h. Whereas, when the gas pressure was 0.06 MPa adhesion in nanofibers occurred because of insufficient pressure elongating the jet. Higher gas pressure facilitated fiber stretching and provided a stronger shearing force between the gas and solution interface for gas cavity when all of the other variable parameters were held constant, and thus resulted in a decrease in fiber diameters. At a lower gas pressure, conversely, the solution jet could not be effectively stretched and the solvent could not sufficiently evaporate, led to fibers agglomeration and adhesion. It was noted that higher gas pressure could result in the waste of gas and excessive turbulence. Additionally, the turbulent air flow at 0.12 MPa made some nanofibers fly out of the range of collector. Thus the optimal gas pressure in this study was determined to be 0.08 MPa.

#### 2.1.4. The Effect of Nozzle Size

**Figure 4:**
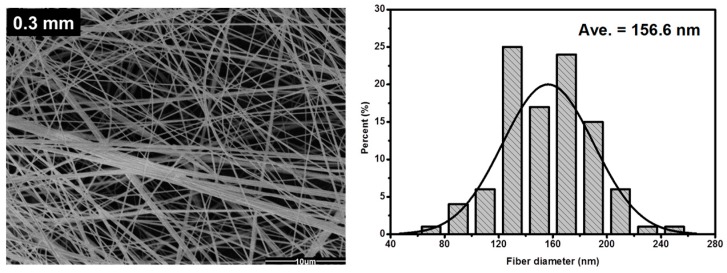

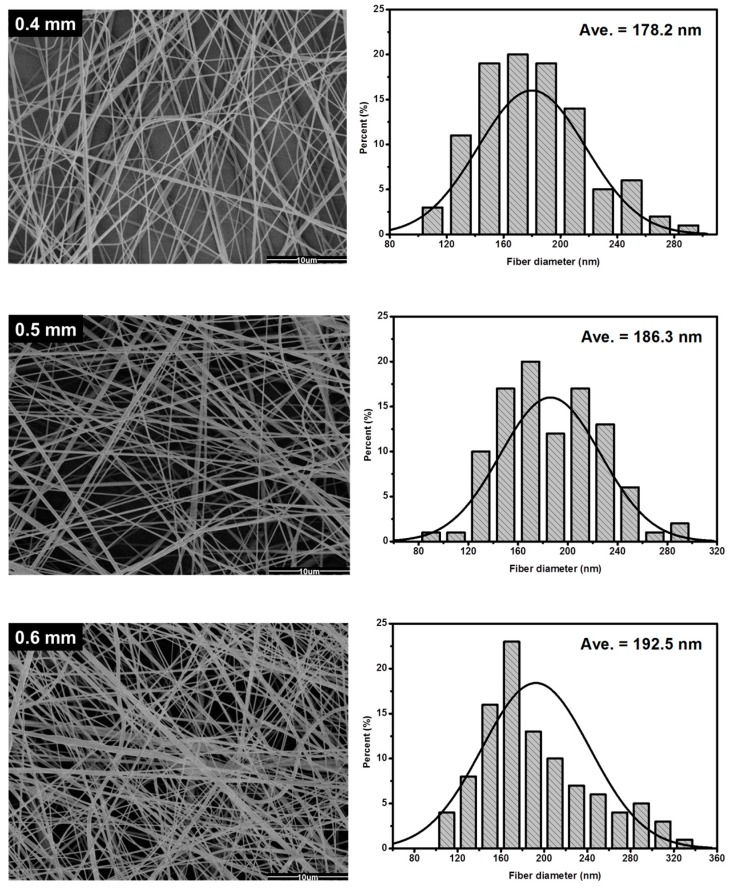
SEM micrographs and diameter distributions of solution blown PAA nanofibers at different nozzle diameters. PAA concentration, solution feeding rate, gas pressure and receiving distance were fixed as 8 wt %, 2.4 mL/h, 0.08 MPa and 20 cm, respectively.

To evaluate the effect of spinneret nozzle size on nanofiber morphology and diameters, four different coaxial nozzles were adopted by varying inner nozzle diameters from 0.3 to 0.6 mm at fixed outer nozzle size and SBS parameters. SEM micrographs and fiber diameter distributions of PAA nanofibers were presented in Figure 4. The results indicated that an increased nozzle diameter led to higher fiber diameters. For the nanofibers got with nozzle diameter of 0.3, 0.4, 0.5, and 0.6 mm, their average diameters were 156.6, 178.2, 186.3, and 192.5 nm, respectively. The variation in the average diameter can be interpreted that when the solution feeding rate remains constant, the diameter of solution stream extruded through larger nozzle became thicker, thus the fibers diameter increased with the increase of nozzle diameter. Meanwhile, the extruding velocity of solution decreased at a larger nozzle size, thus the relative velocity between the gas and the solution increased because the gas flow remained the same. Therefore, the growth trend of fiber diameters slowed down when the nozzle diameter increased from 0.4 to 0.6 mm. Apparently, when the inner diameter of the spinneret was 0.6 mm, the overall morphology of the nanofibers was poor, and there were a mass of junctions and bundles of fibers. Based on this, the optimal inner diameter in this study was determined to be 0.4 mm.

#### 2.1.5. The Effect of Receiving Distance

**Figure 5:**
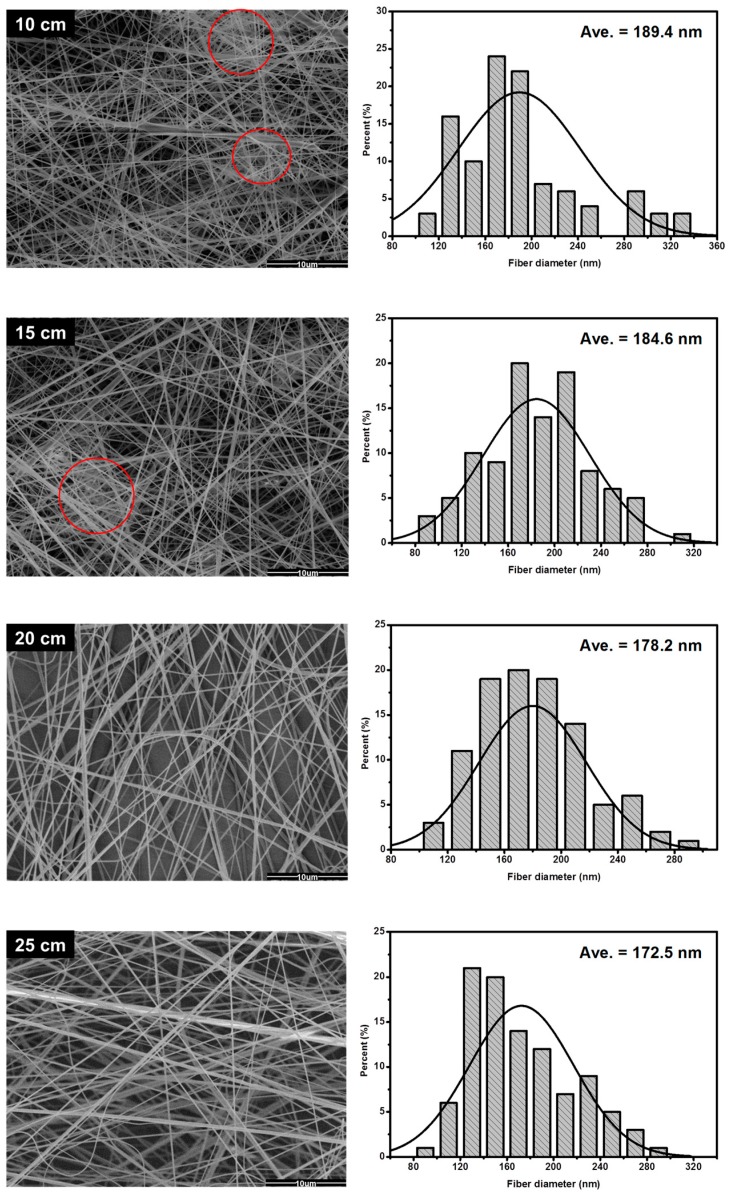
SEM micrographs and diameter distributions of solution blown PAA nanofibers at different receiving distance. PAA concentration, solution feeding rate, gas pressure and inner nozzle diameter were fixed as 8 wt %, 2.4 mL/h, 0.08 MPa and 0.4 mm, respectively.

The so-called receiving distance in SBS process generally means the distance from the nozzle orifice to the surface of the receiving drum, it determines the volatilization time of solvent and the moving distance of the solution jet before it become solidified. Following the optimization of polymer concentration, solution feeding rate, gas pressure, and nozzle size, the effect of receiving distances variable from 10 to 25 cm was discussed in our experiment. As demonstrated in Figure 5, the receiving distance significantly affected the fiber morphology. When the receiving distance was too short (10 and 15 cm), the solution jet struck the collector surface fleetly, accompanied by a lot of non-volatile solvents, the fibers did not have adequate time to be fully dried before arriving at the collector, it appeared to form film-like structure membranes consisting of a mixture of fibers and solutions, as shown circled in red in Figure 5. With the increasing of the receiving distance, it promoted the disappearance of solution droplets and the overall fiber morphology gradually improved. However, increased receiving distance beyond the optimal range (over 30 cm) reduced the amount of nanofibers collected over the identical period. The jet fibers began to fly to every direction, quite a portion of nanofibers could not reach and flied out of the range of collector. Additionally, as seen from Figure 5, with the increase of the receiving distance, the average fiber diameter gradually decreased from 189.4 to 172.5 nm. This mainly depended on the duration of the solvent volatilization, exactly, whether extruded solution streams were stretched to ultrathin jets adequately before they reached on the surface of the collector. The optimal receiving distance in this study was determined to be 20 cm.

### 2.2. Fourier Transform Infrared Spectroscopy (FTIR) Characterization of PAA and PI Nanofibers

**Figure 6:**
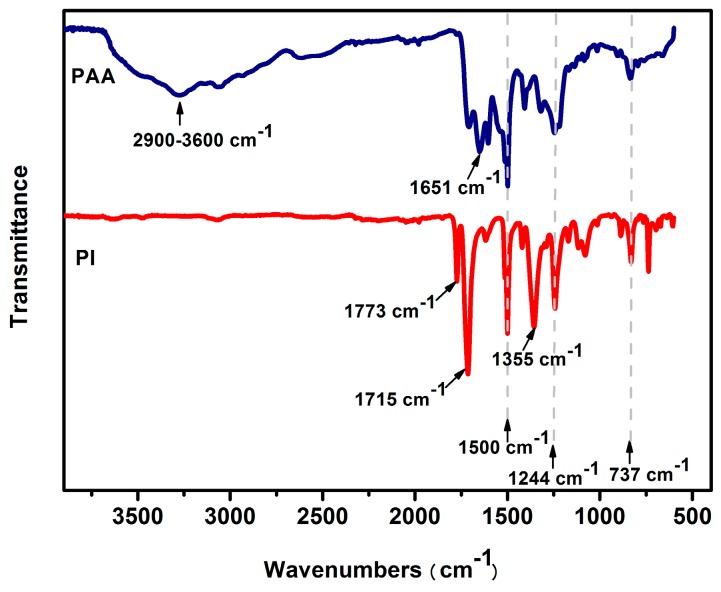
Fourier transform infrared spectroscopy (FTIR) spectrum of nanofibers before and after imidization.

To accurately confirm the conversion from PAA to PI, FTIR was used to characterize the chemical structures of the nanofibers before and after thermal imidization. As presented in Figure 6, both of the spectrums exhibited the characteristic absorption peaks at 1500 and 837 cm^−1^, which were attributed to the C=C stretching and C–H bending vibration in benzene ring of PAA and PI, respectively. The most obvious difference in the FTIR spectrums was the disappearance of the broad absorption peak at 2900–3600 cm^−1^ after thermal imidization, which was attributed to the stretching vibration of carboxyl groups and amide groups of PAA. Meanwhile, the characteristic absorption peak at 1651 cm^−1^ (C=O stretching vibration in –CONH– and –COOH– groups) and 1604 cm^−1^ (N–H bending vibration in –CONH–) of PAA also vanished in PI spectrum, while new characteristic absorption peaks appeared at 1773, 1715, and 1355 cm^−1^ of the PI spectrum, which were accounted for the C=O asymmetric stretching, C=O symmetrical stretching, and C–N stretching in the imide ring, respectively. The above information demonstrated that the PAA nanofibers had been effectively converted to the corresponding PI nanofibers by the thermal imidization process. Furthermore, the absorption peak at 1244 cm^−1^ that appeared in both spectrums was attributed to the stretching of C–O–C, which verified the existence of flexible 4,4′-oxydianiline (ODA) units in the polyamide acid and polyimide molecular chain [34].

### 2.3. X-ray Diffraction of Crystallization, Morphological Structure and Mechanical Properties of PI Nanofibers

**Figure 7:**
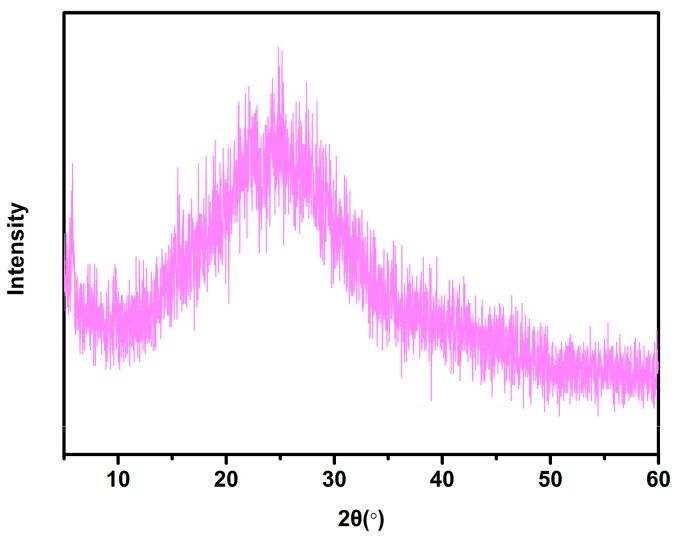
Wide angle X-ray diffraction pattern of polyimide (PI) nanofiber membrane.

Wide angle X-ray diffraction (XRD) was performed for examining the crystallization in the nanofibers (nonwoven membrane made up of PI nanofibers), and the XRD pattern was shown in Figure 7. It can be observed that a wide dispersion peak occurred at about 2*θ* = 24.7°, indicating that a certain ordered crystalline structure has been formed during the solidifying and imidizing process of PAA. The degree of crystallinity of PI nanofibers was 23.56%, which was higher than that of most as-spun PI fibers [35] due to the flexibility of ODA units in PI molecules. The crystallinity of PI nanofibers fabricated by SBS method might contribute to their strong mechanical properties.

**Figure 8:**
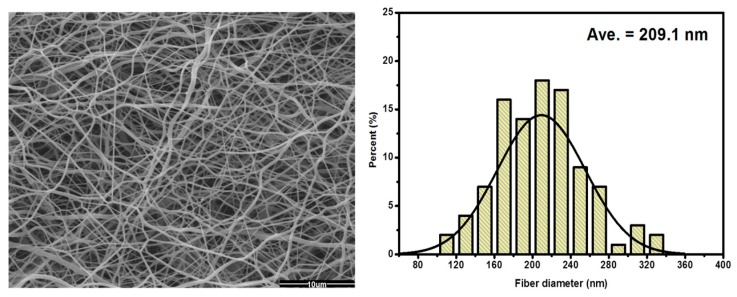
SEM micrograph and diameter distribution of PI nanofibers imidized from PAA nanofibers prepared at the optimal solution blow spinning (SBS) parameters.

Figure 8 showed the SEM image and diameter distribution of PI nanofibers, which was imidized from PAA nanofibers that were prepared at the optimal SBS parameters. Noticeably, straight PAA nanofibers deformed after imidization, these PI nanofibers presented adhesion morphology among the interconnected fibers with a relatively smooth appearance. This phenomenon might be attributed to the presence of ODA flexible segments, which probably led to the nanofibers deformation in thermal imidization process. Fortunately, this feature results from the intersection of nanofibers forming mats could favor the enhancement of mechanical properties.

**Figure 9:**
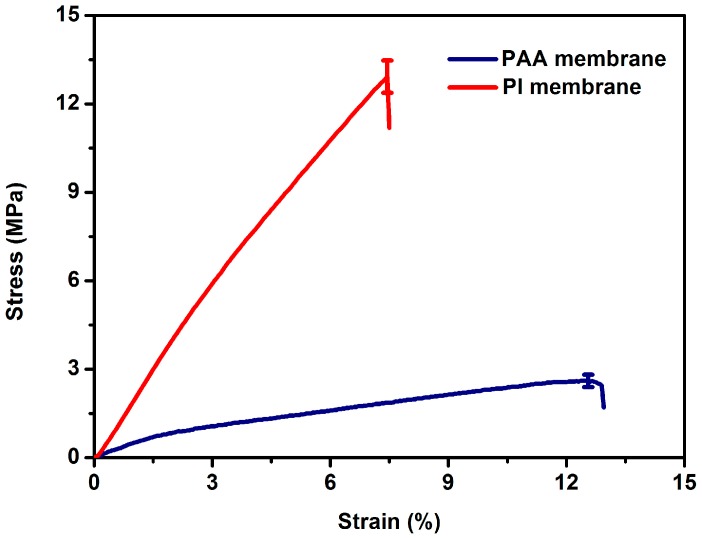
Tensile stress-strain curves of PAA and PI nanofiber membranes.

The imidization of PAA is considered to be chemical change accompanied by a molecular chain self-ordering process. As shown in Figure 9, the tensile strength of the nanofiber mat increased from 2.60 (±0.21) to 12.95 (±0.55) MPa after thermal imidization, this was attributed to not only chemical change from PAA to PI, but also to the interconnection of nanofibers after thermal imidization and their high crystallinity (as shown in Figure 7). On the other hand, the diameter of PI nanofibers exhibited a significant increase in diameter with an average diameter of 209.1 nm (Figure 8), when compared to that of PAA nanofibers. Generally, the diameter of PI nanofibers could slightly reduce on account of the escape of water molecules after imidization, nevertheless, this phenomenon might ascribed to the fiber shrinkage and adhesion caused by fiber deformation and amalgamation.

## 3. Materials and Methods 

3,3′,4,4′-Biphenyltetracarboxylic dianhydride (BPDA, ≥99.7%) and 4, 4′-oxydianiline (ODA, ≥99.6%) were supplied by Changzhou Sunlight Pharmaceutical Co., Ltd., (Changzhou, China). p-Phenylenediamine (PDA) was purchased from Sigma-Aldrich (Shanghai, China). *N*,*N*-Dimethylformamide (DMF) with analytical grade was provided by Shanghai Ling Feng Chemicals Co., Ltd., (Shanghai, China) and stored over 4 Å activated molecular sieves for at least a week prior to use.

### 3.1. Synthesis of Precursor PAA Solution

PAA for spinning was synthesized from BPDA/PDA/ODA with molar ratio of 2:1:1. The synthesis process was as follows: 1.080 g (0.01 mol) PDA and 2.002 g (0.01 mol) ODA were first dispersed in DMF within a three-neck flask under dry nitrogen gas. After the diamine was almost dissolved, 5.94 g (0.02 mol) BPDA was gradually added with four batches in an hour. The polycondensation process was performed with intense stirring at 0 °C for 24 h. A pristine PAA solution with a concentration of 13 wt % was obtained eventually. The inherent viscosity of PAA solution was measured to be 3.56 dL/g by diluting a bit precisely weighted amount of the as-synthesized PAA solution with DMF. The obtained PAA solution was stored in refrigerator for later use.

### 3.2. The Preparation of PAA Nanofibers via SBS

**Figure 10:**
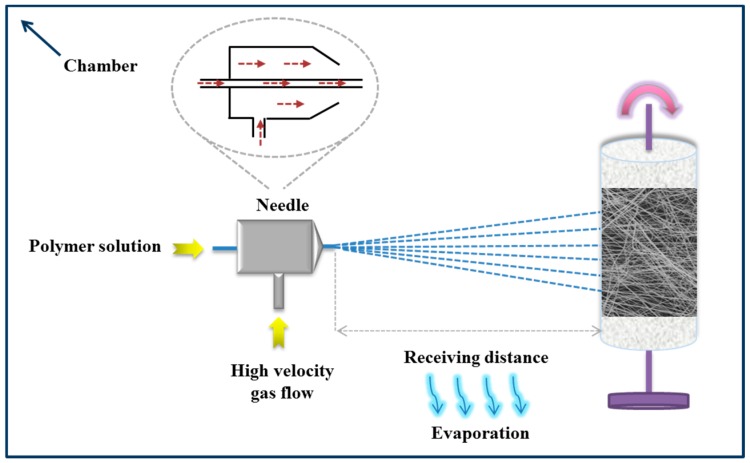
The schematic of self-made SBS apparatus.

A special-designed SBS apparatus used for the experiments was schematically shown in Figure 10. The self-made parallel coaxial nozzles (PAA solution flows through the inner nozzle, and a source of pressurized gas flows through the outer nozzle) were in the enlarged schematic. In brief, PAA solution was injected through the inner nozzle by a syringe pump (LongerPump, LSP01-1A, Baoding, China), meanwhile a highly pressurized gas flow as fiber forming driving force was released through the outer nozzle. As soon as the PAA solution stream was pressed out of the nozzle tip, the formed solution droplet was simultaneously blown and attenuated into ultrathin jets by high-velocity gas flow (90–111 m/s), accompanied by carrier solvent that was volatilized during fixed receiving distance between the nozzle and collector, the solidified nanofibers were deposited on the collector surface. It is worth mentioning that the pressurized gas should be inert gases, such as argon, nitrogen, etc. In the present work, the pressurized gas used was purity nitrogen. In addition, we equipped two infrared heating lamps along the spinning line (between the nozzle and the collector) to heat the air in order to speed up the evaporation of the solvent, the ambient temperature was about 60 °C.

The as-prepared PAA solution was diluted to different concentrations with a calculated amount of DMF; they were magnetically stirred for 10 h to ensure homogeneity. SBS process was carried out as follows: the homogeneous PAA solution was loaded into a 20 mL disposable syringe that was connected with the coaxial nozzle, and was extruded through the inner nozzle by a syringe pump at a certain rate (varied from 0.8 to 3.2 mL/h). The pressurized gas source was controlled with a pressure regulator, and the gas pressure varied according to experiment design. The inner nozzle was protruded 4.5 mm beyond the concentric outer nozzle. The blow-spun PAA nanofibers were deposited on a rotating drum as a collector, which was covered with aluminum foil, the drum was positioned at a fixed distance from the nozzle and the rotational speed of the collector was maintained at 150 rpm. After being spun for a certain time, the collected PAA nanofiber mat was peeled off carefully from the foil, and was subsequently dried in a vacuum oven at 60 °C overnight to volatilize the residual solvent.

### 3.3. Conversion from PAA Nanofibers to PI Nanofibers

**Scheme 1:**
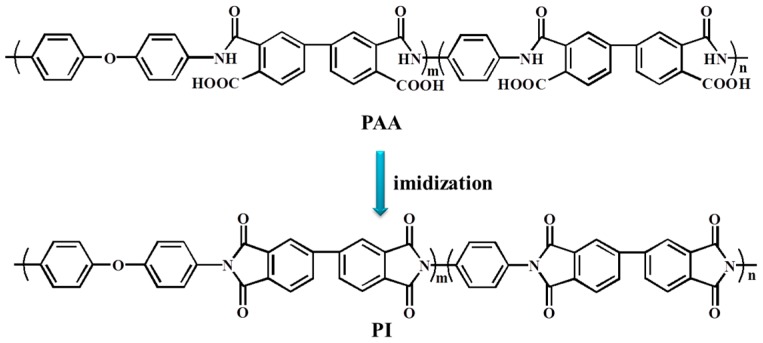
The molecular structural formula of PAA and PI.

The thermal imidization of the as-spun PAA nanofibers was performed in a high temperature pipe furnace under argon atmosphere. The procedure was set as follows: heating up to 250 °C at a rate of 5 °C /min and annealing for 1 h, then heating up to 340 °C at a rate of 1 °C/min and annealing for 1 h, and finally cooling down to room temperature. The molecular structural formula of PAA and PI was shown in Scheme 1.

### 3.4. Characterization

Surface morphological observations of solution blown PAA were performed with a field emission scanning electron microscope (FE-SEM, SU8010, Hitachi, Japan) after sputter-coated with 5–10 nm of gold. Fiber diameters were determined using Image Tool software (Texas City, TX, USA) by randomly selecting 100 individual fibers per specimen from at least five different SEM images with 5000× magnification. The structure of the PAA and PI membrane were confirmed by fourier transform infrared spectroscopy (FT-IR, NICOLET NEXUS-670, Waltham, MA, USA) with the attenuated total reflectance (ATR) mode. All spectra were run in 32 scan per spectrum in the range of 4000–600 cm^−1^ with the resolution of 0.09 cm^−1^. A wide angle X-ray diffractometer (XRD, D/max-2550 PC, Rigaku Co., Akishima, Japan) with a CuKα (*λ* = 1.5406 Å) was used to confirm the crystallographic structure of the sample. The diffraction angle 2*θ* was recorded from 5° to 60°. The tensile tests of as-prepared membranes were performed on a tensile tester (XQ-1C, Shanghai New Fiber Instrument Co., Ltd., Shanghai, China) at a crosshead speed of 10 mm/min in ambient condition. The gauge length was 10 mm and the dimensions of test specimens were 3 mm wide and 25 μm thick. Every series of samples were measured ten times.

## 4. Conclusions

In summary, we succeeded in fabricating PAA nanofibers by a novel solution blow spinning method and then imidized them into PI nanofibers. The effect of solution blow spinning parameters on the morphology and diameter distributions of PAA nanofibers was discussed. It was found that solution concentration exhibited a notable correlation with spinnability and the formation of bead defects in PAA nanofibers. Accordingly, other parameters, including solution feeding rate, gas pressure, nozzle size, and receiving distance influenced the morphology and diameter distribution of PAA nanofibers. It was revealed that uniform PAA nanofibers were got under the optimum process parameters of 8 wt % PAA concentration, 2.4 mL/h solution feeding rate, 0.08 MPa gas pressure, 0.4 mm inner nozzle, and the receiving distance of 20 cm. The diameters of resultant PAA nanofibers distributed from 130 to 210 nm with an average value of 178.2 nm. FTIR confirmed that PAA nanofibers were effectively converted to PI nanofibers by the thermal imidization process. Furthermore, the XRD pattern indicated the degree of crystallinity of PI nanofibers was 23.56%, which contributed to the nanofibers’ strong mechanical properties. The PI nanofibers exhibited obvious adhesion morphology among interconnected fibers, along with the average diameter of 209.1 nm and tensile strength of 12.95 MPa, which was increased significantly when compared to that of PAA nanofiber mat (2.60 MPa). This research provides a novel and effective method to fabricate PI nanofiber mats.
